# Selective salvage of zones 2 and 4 in the pedicled TRAM flap: a focus on reducing fat necrosis and improving aesthetic outcomes

**DOI:** 10.1186/s40064-016-1714-7

**Published:** 2016-01-22

**Authors:** Chi Sun Yoon, Kyu Nam Kim

**Affiliations:** Department of Plastic and Reconstructive Surgery, Ulsan University Hospital, University of Ulsan College of Medicine, 877 Bangeojinsunhwando-ro, Dong-gu, Ulsan, 682-714 Korea

**Keywords:** Breast reconstruction, Rectus abdominis, Pedicled flap, TRAM

## Abstract

The introduction of microsurgery has decreased the frequency of using the pedicled transverse rectus abdominis musculocutaneous (TRAM) flap, owing to its higher incidence of fat necrosis and limitations in flap insetting (vs. the free TRAM flap). We devised an efficient method of using zones 2 and 4, based on the pedicled flap’s vascular anatomy, to reduce fat necrosis and achieve superior aesthetic outcomes during immediate breast reconstruction using the pedicled TRAM flap. Between April 2011 and February 2015, we performed immediate breast reconstruction using the contralateral unipedicled TRAM flap for 136 breast cancer patients. The conventional method (insetting by removing zone 4 and part of zone 2) was used for 83 patients (Group A), and selective salvage of zones 2 and 4 (our proposed method) was used for 53 patients (Group B). Fat necrosis was observed in 17 patients (20.48 %) and 4 patients (7.55 %) from Groups A and B, respectively (p = 0.033). The average patient satisfaction scores at 6 months (comparing the reconstructed and contralateral breasts) were 7.01 and 8.02 in Groups A and B, respectively (p < 0.001). Liposuction to improve the upper-pole contour was performed at 6 months for 13 patients in Group A, although no patients in Group B required a secondary procedure (p = 0.002). Fat necrosis can be reduced via selective salvage of zones 2 and 4, based on the pedicled TRAM flap’s vascular anatomy. Furthermore, superior aesthetic outcomes can be achieved via flap insetting using the three-dimensional concept. *Level of evidence* Procedure comparison, Level II.

## Background

Since breast reconstruction using the transverse rectus abdominis musculocutaneous (TRAM) flap was introduced by Hartrampf et al. (1982), the pedicled TRAM flap has been widely used for breast reconstruction. However, recent improvements in microsurgery techniques have led to the increased use of the free TRAM flap, in order to reduce fat necrosis (a complication that is associated with flap perfusion), and the frequency of using the pedicled TRAM flap is gradually decreasing (Kim et al. 2007). Fat necrosis is one of the most common complications of breast reconstruction using the TRAM flap, and is caused by ischemic necrosis in the subcutaneous fat. This fat necrosis presents as a firm area at the periphery of the flap, and is diagnosed using palpation or mammography (Kim et al. 2007). The fat necrosis can have various causes, including fixation suturing of the flap, smoking, extensive use of the flap, obesity, and postoperative radiation therapy, although the most important cause is inadequate blood supply (Kim et al. 2007). When Hartrampf et al. (1982) described the pedicled TRAM flap, they indicated that the abdominal skin ellipse of the flap was divided into four zones, based on the vascular anatomy. In this context, the blood supply is lowest in zone 4 (the distal contralateral zone). Therefore, zone 4 is generally removed intraoperatively, and the distal area of zone 2 (the proximal contralateral zone) is also removed in many cases, to reduce the risk of fat necrosis (Kim et al. 2007; Moon and Taylor 1988; Kanchwala and Bucky 2008a; Yamaguchi et al. 2004; Kotti 2014). In this study, we describe a method to efficiently use zones 2 and 4, based on the flap’s vascular anatomy, in order to prevent fat necrosis and achieve superior aesthetic outcomes during immediate breast reconstruction using the pedicled TRAM flap.

## Methods

All examinations and procedures in the present study were approved by our institutional review board (UUH 2015-04-020), and informed consent was obtained from all patients. Although the revised Hartrampf classification of perfusion zones (zones 1 and 2 as ipsilateral and zones 3 and 4 as contralateral) is usually used for free TRAM flaps, we applied the original Hartrampf classification of perfusion zones (zone 1, ipsilateral proximal; zone 2, contralateral proximal; zone 3, ipsilateral distal; and zone 4, contralateral distal) for the pedicled TRAM flap. Between April 2011 and February 2015, we performed immediate breast reconstruction using the contralateral unipedicled TRAM flap after performing skin-sparing mastectomy or nipple-areolar complex-sparing mastectomy in 136 breast cancer patients. Between 2011 and 2013, 83 patients (Group A) underwent flap insetting by removing zone 4 and part of zone 2 (the conventional method). Between 2014 and 2015, 53 patients (Group B) underwent flap insetting via selective salvage of zones 2 and 4 (our proposed method). In Group A, the mean age was 46.89 ± 6.82 years (range 29–59 years), the mean body mass index (BMI) was 22.42 ± 1.73 kg/m^2^ (range 18.31–25.76 kg/m^2^), and the mean follow-up period was 24.58 ± 6.09 months (range 16–28 months). In Group B, the mean age was 45.96 ± 5.98 years (range 35–58 years), the mean BMI was 22.42 ± 1.74 kg/m^2^ (range 17.64–26.61 kg/m^2^), and the mean follow-up period was 23.66 ± 5.52 months (range 15–24 months) (Table 1). A diagnosis of fat necrosis was made when a mass that was firmly palpable (without loss of skin tissue in the reconstructed breast) persisted for >6 months and was confirmed via ultrasonography.

### Operative techniques

**Fig. 1:**
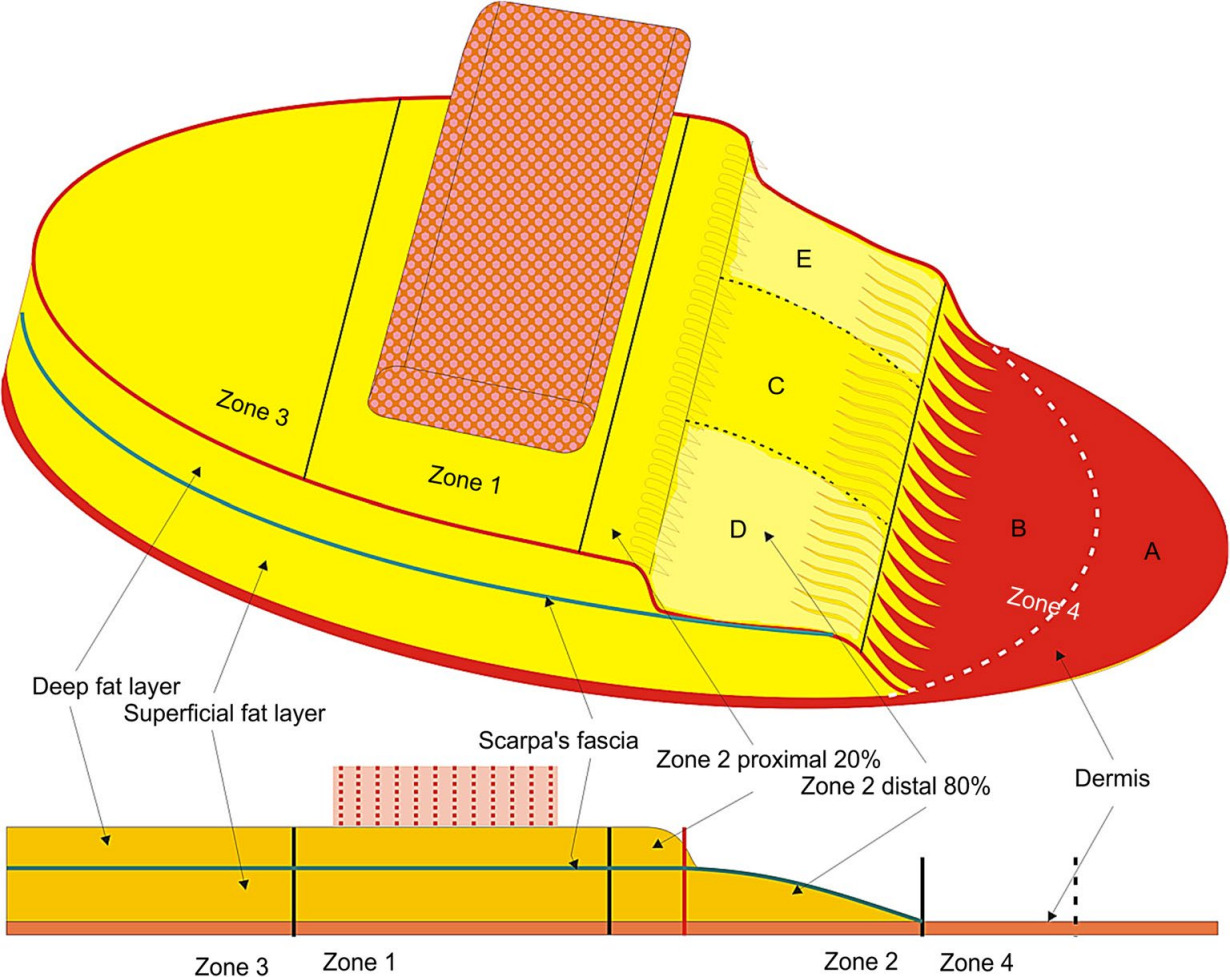
A schematic of the selective salvage of zones 2 and 4 in the unipedicled transverse rectus abdominis musculocutaneous (TRAM) flap. **a** The distal area of zone 4 is discarded. **b** The proximal area of zone 4 is formed into a vascularized dermis graft to enhance the peripheral contour of the upper pole and to prevent fat necrosis. **c** The middle area of the distal 80 % of zone 2 is formed into a dermo-adipofascial flap, which includes the dermis and thick superficial fat layer (with Scarpa’s fascia). **d**, **e** The *upper* and *lower areas* of the distal 80 % of zone 2 are formed into a dermofat flap, which includes the dermis and thin superficial fat layer. In this manner, by selective salvage of zones 2 and 4, TRAM flap fills the volume more naturally at the upper pole of the breast skin envelope, and prevents fat necrosis by focusing on the deep fat layer

**Fig. 2:**
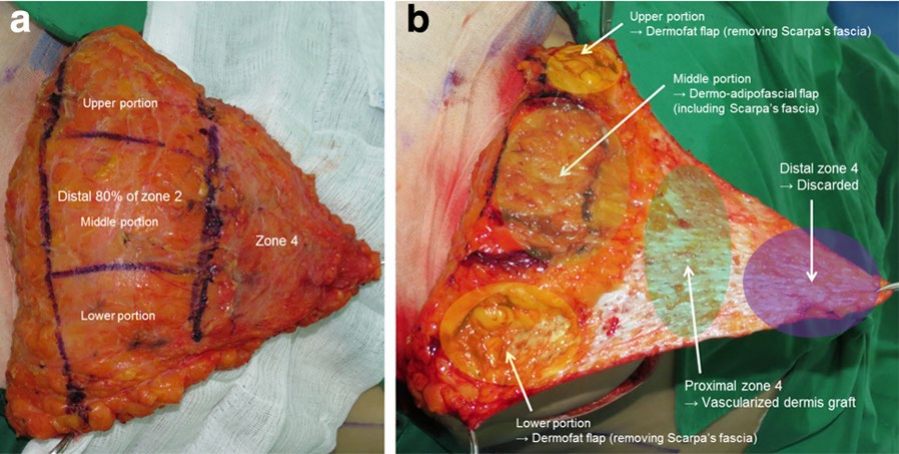
Intraoperative findings. Upon discarding the distal part of zone 4, Scarpa’s fascia and the superficial fat tissue in the proximal part of zone 4 are entirely removed, leaving only the dermal tissue. Thus, the proximal part of zone 4 is formed into a vascularized dermis graft. The *upper* and *lower portions* of the distal 80 % of zone 2 are used to form a dermofat flap (including the dermis and thin superficial fat tissue) by removing Scarpa’s fascia and part of the superficial fat tissue. The *middle portion* of the distal 80 % of zone 2 is used to form a dermo-adipofascial flap (including the dermis and thick superficial fat tissue, and retaining Scarpa’s fascia)

All reconstructive procedures were performed by a single surgeon (the senior author). In the proposed method, immediate breast reconstruction was performed using selective salvage of zones 2 and 4 in superiorly based contralateral unipedicled TRAM flaps, after skin-sparing mastectomy or nipple-areolar complex-sparing mastectomy. Based on Hartrampf’s concept of the conventional zones for TRAM flaps, we marked zones 1–4 after transposing the flap from the abdomen to the breast. After de-epithelializing zones 2 and 4, all deep fat tissue under Scarpa’s fascia was removed from the distal 80 % of zone 2 and all of zone 4. For zone 4, after discarding the distal area, Scarpa’s fascia and the superficial fat tissue in the proximal area were entirely removed, which left only the dermis. The upper and lower portions of the distal 80 % of zone 2 were used to form a dermofat flap (including the dermis and thin superficial fat tissue) by removing Scarpa’s fascia and part of the superficial fat tissue. The middle portion of the distal 80 % of zone 2 was used to form a dermo-adipofascial flap (consisting of the dermis and thick superficial fat tissue, while retaining Scarpa’s fascia). In these defatting procedures, it is important to confirm the fat tissue’s viability via good visible bleeding after the resection. Zones 1 and 3 and the proximal 20 % of zone 2 were used to preserve all the layers of the flap (Figs. 1, 2).

**Fig. 3:**
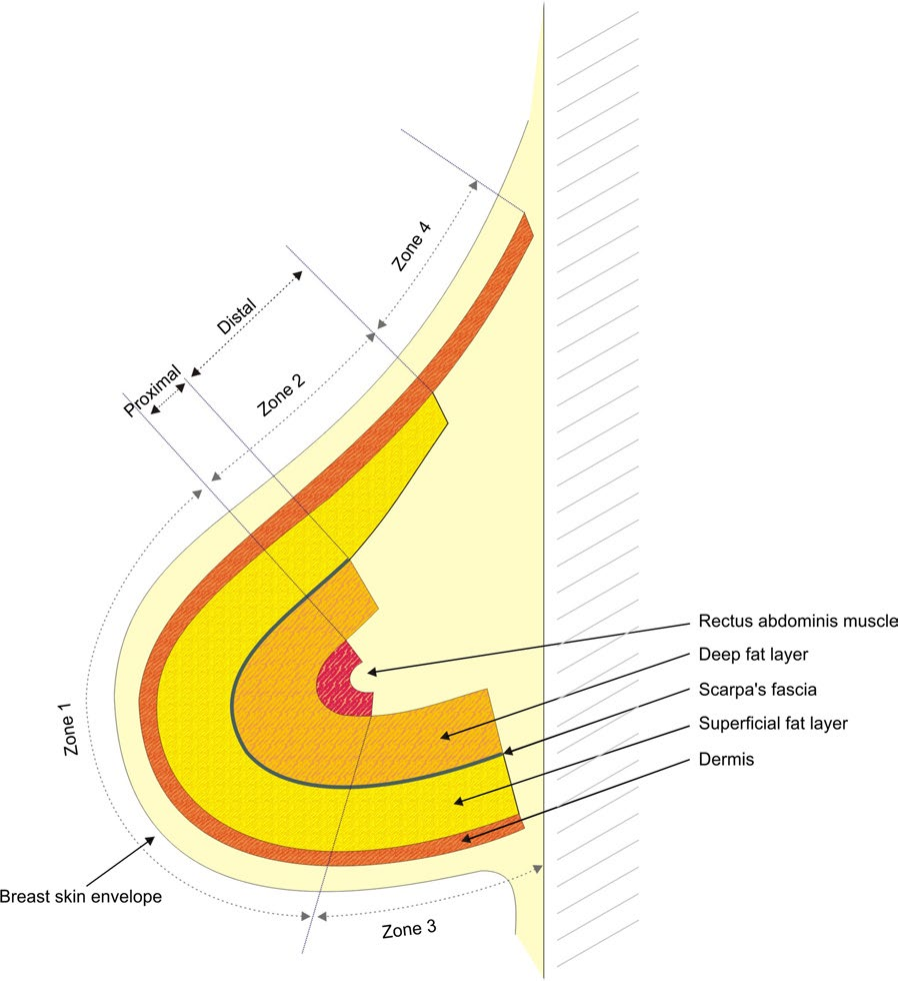
A schematic representation of the transverse rectus abdominis musculocutaneous flap insetting into the breast skin envelope. The proximal area of zone 4 forms the vascularized dermis graft, and lies on the periphery of the upper pole. The distal 80 % of zone 2 forms the dermo-adipofascial flap in the middle area and the dermofat flap in upper and lower area, and lies on the central area of the upper pole. Zone 1 and the proximal 20 % of zone 2 lie in the middle pole, and zone 3 lies in the lower pole

For flap insetting, we used the vertical or oblique insetting method (angle, 45°–90°), in which zone 3 was positioned in the lower pole, and the proximal area of zone 4 was positioned in the upper pole. After dividing the breast into the upper, middle, and lower poles, the proximal area of zone 4 was positioned as a vascularized dermis graft in the periphery of the upper pole. To prevent spontaneous rotation and displacement of the flap, we placed a fixation suture to attach the pectoralis major muscle and the proximal area of zone 4, and positioned it in the periphery of the upper pole. In the center of the upper pole, the distal 80 % of zone 2 was positioned as dermofat and dermo-adipofascial flaps. The proximal 20 % of zones 1 and 2 were positioned in the middle pole, and zone 3 was positioned in the lower pole (Fig. 3).

### Statistical analysis

Continuous variables were expressed as mean and standard deviation, and categorical variables were expressed as frequency and percentage. Student’s t test (continuous variables) and Fisher’s exact test (categorical variables) were used to compare differences between the two methods (Group A vs. Group B). All statistical analyses were performed using SPSS software (version 21; SSPS Inc., Chicago, IL), and the significance level was set at a p value of <0.05.

## Results

When we compared Groups A and B, there was no significant difference in age (p = 0.418) or BMI (p = 0.983) (Table 1).

**Table 1:** A comparison of the conventional method (Group A) and proposed method (Group B)

	Group A (n = 83)	Group B (n = 53)	*P* value
Mean age (years)	46.89 ± 6.82	45.96 ± 5.98	0.418
Mean BMI (kg/m^2^)	22.42 ± 1.73	22.42 ± 1.74	0.983
Fat necrosis (n)	17/13^¥^ (20.48 %)	4/3^¥^ (7.55 %)	0.033
Extent of fat necrosis	10–30 % of zone 2	10–20 % of the distal 80 % of zone 2	N/A
Mean patient satisfaction scores	7.01 ± 0.94	8.02 ± 0.82	<0.001
Secondary touch-up procedures in the upper pole	13 (15.66 %)	0	0.002

*BMI* body mass index

^¥^Number of patients with a history of smoking

### The conventional method (Group A; removal of zone 4 and part of zone 2)

In Group A (n = 83), fat necrosis was observed in 17 patients (20.48 %). Twelve patients developed fat necrosis in the distal area of zone 2, and 5 patients developed fat necrosis in the proximal and distal areas of zone 2. A history of smoking in the previous 6 months was observed for 9 patients with fat necrosis in the distal area of zone 2, and for 4 patients with fat necrosis in the proximal and distal areas of zone 2. The extent of the fat necrosis varied from 10 to 30 % of zone 2. For patients with fat necrosis, fat necrosis removal was performed at 6 months postoperatively using local anesthesia and via the direct approach or previous scar line. Patient satisfaction was assessed at 6 months, using a scale of 1–10 to compare the reconstructed and contralateral breasts in terms of volume, contour, and the level of the infra-mammary fold. The mean patient satisfaction score was 7.01 ± 0.94 (range 5–9). During the outpatient follow-up at 6 months, we performed liposuction for 13 patients to improve the upper-pole contour of their reconstructed breast (Table 1).

### The proposed method (Group B; selective salvage of zones 2 and 4)

In Group B (n = 53), fat necrosis was observed in 4 patients (7.55 %). Three patients developed fat necrosis in the upper portion of the distal 80 % of zone 2 (the upper medial area of the reconstructed breast), and 1 patient developed fat necrosis in the lower portion of the distal 80 % of zone 2 (the upper lateral area of the reconstructed breast). No patients developed fat necrosis in the proximal area of zone 4 (in the periphery of the upper pole). One patient with fat necrosis in the upper portion of the distal 80 % of zone 2 was an active smoker, and 2 patients had a history of smoking within 6 months. The patient with fat necrosis in the lower portion of the distal 80 % of zone 2 had a previous Caesarean section scar. The extent of the fat necrosis was 20 % of the distal 80 % of zone 2 among the active smokers with fat necrosis in the upper pole, compared to <10 % among the rest of the patients with fat necrosis. Four patients underwent fat necrosis removal at 6 months using local anesthesia and via the direct approach or previous scar line. The mean patient satisfaction score in Group B was 8.02 ± 0.82 (range 6–9). None of the patients required a secondary procedure at the 6-month follow-up (e.g., liposuction or fat injection to improve the upper-pole contour of the reconstructed breast) (Tables 1, 2).

**Table 2:** Data from patients who developed fat necrosis after undergoing the proposed method

Case	Age (years)	Extent of fat necrosis area	Risk factor
6	47	10 % in lower portion of the distal 80 % of zone 2	Previous Caesarean section scar
11	42	10 % in upper portion of the distal 80 % of zone 2	Smoking history
23	45	10 % in upper portion of the distal 80 % of zone 2	Smoking history
47	51	20 % in upper portion of the distal 80 % of zone 2	Active heavy smoker

## Discussion

Breast reconstruction methods using autologous abdominal tissue have been developed to maximize the vascular perfusion of the flap, in order to reduce the incidence of partial flap loss, fat necrosis, and donor site morbidity. Based on the improvements in microsurgical techniques, a large proportion of breast reconstruction procedures have been performed using the free TRAM flap, free deep inferior epigastric perforators, and free superficial inferior epigastric artery flaps. However, these free flap techniques require skilled microsurgeons, as well as specially educated doctors and nurses to perform postoperative flap monitoring, and the risk of total flap loss should be considered when attempting these techniques. Thus, the use of free flap techniques may be limited, depending on the center’s clinical setting (Kotti 2014; Kanchwala and Bucky 2008a). In contrast, breast reconstruction using the pedicled TRAM flap has a relatively easy learning curve (vs. the free flap), does not require special postoperative flap monitoring, and is a very stable procedure, with a flap survival rate of up to 99.8 % (Kim et al. 2009). However, the pedicled TRAM flap has a higher incidence of fat necrosis, compared to free flap techniques (Kim et al. 2007, 2009), and flap insetting can be limited, depending on the pedicle. This may lead to less optimal aesthetic outcomes, compared to those for the free flap techniques. Therefore, we devised a more effective method for processing zones 2 and 4 (selective salvage), based on the vascular anatomy of the pedicled TRAM flap, in order to reduce the incidence of fat necrosis and achieve superior aesthetic outcomes.

When using the pedicled TRAM flap, the lower transverse abdominal island flap is dependent on the deep superior epigastric artery, which is supplied in a retrograde fashion by the deep inferior epigastric artery (Moon and Taylor 1988). The skin paddle is also supplied by a direct perforator from the proximal part of the deep inferior epigastric artery. In the ipsilateral distal portion of the skin flap, the deep inferior epigastric artery perforators are anastomosed with branches of the superficial inferior epigastric artery (Moon and Taylor 1988). Once they cross over the midline, the perforators of the contralateral deep inferior epigastric artery send branches to the subdermal plexus, although there is no vascular filling under Scarpa’s fascia or into the contralateral superficial inferior epigastric artery. After the midline crossover, vascular perfusion of the flap mainly occurs via choke connecting vessels in the surface of the subdermal plexus and rectus sheath (Moon and Taylor 1988; Kotti 2014). Thus, there is no perfusion in the deep fat layer under Scarpa’s fascia when crossing over the midline in zones 2 and 4. Furthermore, if >20 % of zone 2 is used for the pedicled TRAM flap, fat necrosis increases by 2.75-fold (Kim et al. 2007). Therefore, if >20 % of the zone 2 tissue is required, the reconstructive plan should be changed to a bipedicled or free flap (Kim et al. 2007; Shestak 1998).

Based on these considerations, we devised a more selective technique to salvage for zones 2 and 4, which allows us to selectively resect the tissue with the greatest risk of fat necrosis. Thus, we removed the deep fat tissue under Scarpa’s fascia at the distal 80 % of zone 2, and used the upper and lower portions (where perfusion decreases) to form a dermofat flap by leaving a thin layer of the superficial fat tissue. In addition, we used the middle portion to form a dermo-adipofascial flap by retaining Scarpa’s fascia. In a study by Kim et al. (2007), fat necrosis occurred in 65 (14.5 %) of 400 patients who underwent immediate breast reconstruction using the unipedicled TRAM flap, with fat necrosis in zone 2 (54 patients), zone 3 (10 patients), and zone 1 (1 patient). In the present study, we also observed fat necrosis (10–30 % of zone 2) in 17 patients (20.48 %) from Group A. In contrast, only 4 patients (7.55 %) from Group B developed fat necrosis in the distal 80 % of zone 2 (<20 % of this area), and the fat necrosis removal was easily performed at the 6-month follow-up. Furthermore, the incidence of fat necrosis was significantly lower in Group B, compared to that in Group A (p = 0.033). Moreover, the 4 patients with fat necrosis in Group B had extrinsic risk factors, including a smoking history or previous operative scar, which may have affected the occurrence of fat necrosis. Thus, it is possible that our procedure carries a minimal risk of necrosis due to factors that are intrinsic to the TRAM flap. To prevent spontaneous rotation, we fixed the periphery of the flap to the pectoralis major muscle (using fixation sutures) during the flap insetting. However, the use of a fixation suture can disturb the blood supply to the subcutaneous fat, and may allow fat necrosis to occur (Kim et al. 2007, 2009). Therefore, we designed zone 4 as a vascularized dermis graft, by cutting the distal area, leaving the dermis in the proximal area, and removing the superficial and deep fat tissue. This vascularized dermis graft does not have fat tissue, which precludes any possibility of the fixation suture leading to fat necrosis. This approach appeared to be successful, as none of the patients in Group B (our proposed method) developed fat necrosis in the proximal area of zone 4 in the periphery of the upper pole.

**Fig. 4:**
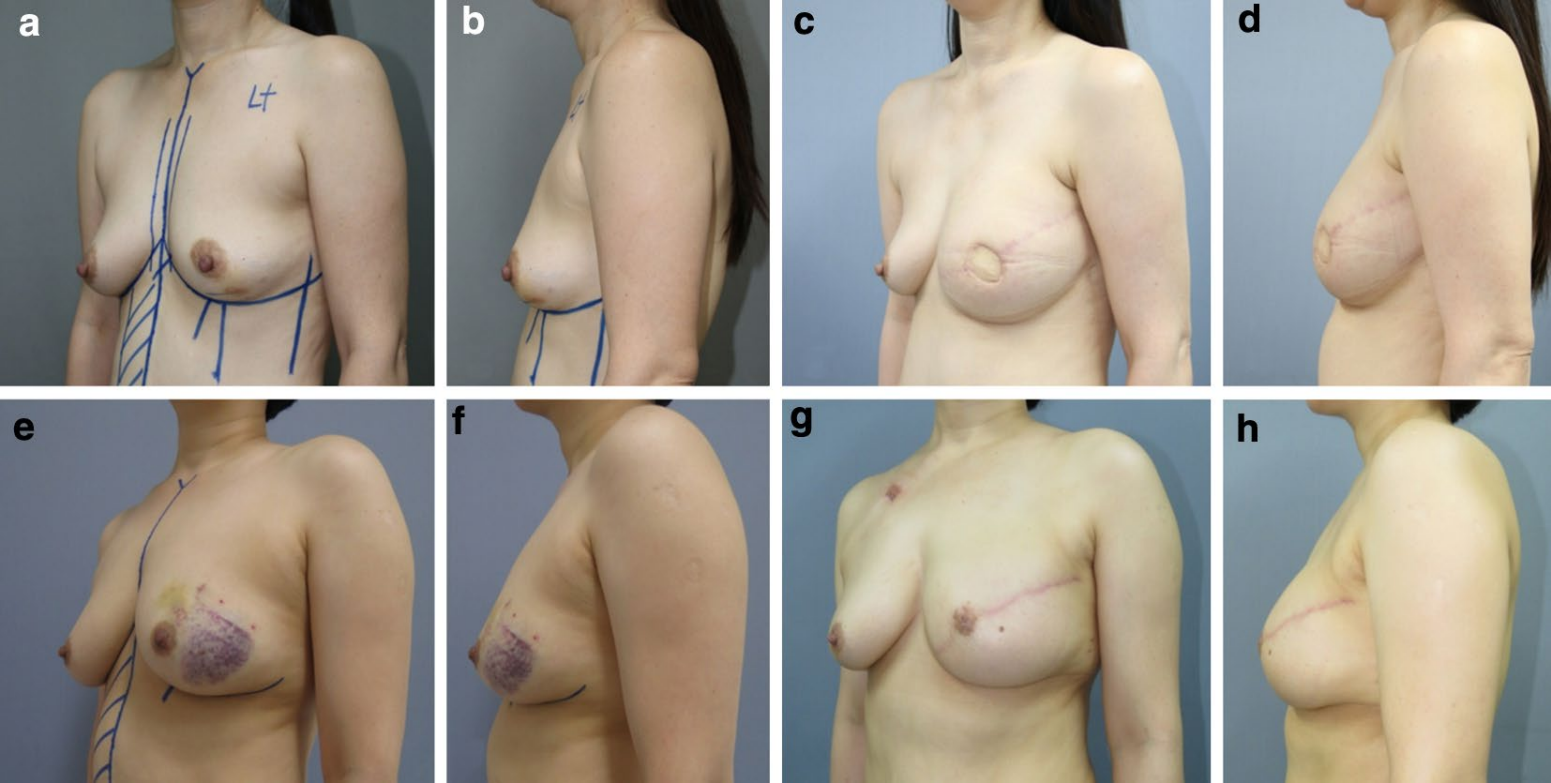
**a**, **b** A 47-year-old patient with left breast cancer is shown with preoperative markings for a left nipple-areolar complex-sparing mastectomy and contralateral pedicled transverse rectus abdominis musculocutaneous (TRAM) flap reconstruction using the proposed method. **c**, **d** Her postoperative results at 17 months show the natural upper-pole contour of the left reconstructed breast. **e**, **f** A 45-year-old patient with left breast cancer is shown with preoperative markings for a left skin-sparing mastectomy and contralateral pedicled TRAM flap reconstruction using the proposed method. **g**, **h** Her postoperative results at 19 months (before nipple reconstruction) show the natural upper-pole contour of the left reconstructed breast

The development and popularization of skin-sparing mastectomy has allowed modern breast reconstruction to achieve important qualitative aesthetic outcomes. For example, a shaping surgery has been replaced by a filling surgery that preserves the vital anatomical landmarks of the breast envelope like infra-mammary crease and boundaries of the breast (Kanchwala and Bucky 2008b). Skin-sparing mastectomy has also made it possible to rebuild the appropriate three-dimensional shape of the reconstructed breast using a two-dimensional pattern from the abdomen (Kanchwala and Bucky 2008b). To achieve the idea three-dimensional breast shape, a straight line is drawn to account for the linear increase in volume from the most upper pole to the nipple-areolar complex, and a convex line is drawn to account for the change from the nipple-areolar complex to the inframammary crease. This process allows us to achieve good aesthetic outcomes after breast reconstruction, by filling up the periphery of the upper pole with a minimum volume, gradually accreting the volume while approaching the center of the upper and middle poles, and finishing naturally in the lower pole. However, the movement of the skin paddle from the pedicled TRAM flap is relatively limited, compared to the relatively free movement of the skin paddle after insetting a free flap. Thus, to achieve superior aesthetic outcomes, flap insetting must carefully considered when using pedicled TRAM flaps. In our proposed method, zone 4 (the thinnest portion of the flap) is positioned at the periphery of the upper pole as a vascularized dermis graft, and the distal 80 % of zone 2 (the second thinnest portion) is positioned in the center of the upper pole as dermofat and dermo-adipofascial flaps. In the center of the upper pole, a slightly thicker dermo-adipofascial flap is placed in the centermost area, and the relatively thin dermofat flap is placed in the periphery. Thus, the whole layer of zone 1 and the proximal 20 % of zone 2 fill the middle pole (which requires the greatest volume), and the whole layer of zone 3 fills the lower pole as a fold, which can provide a natural lower curve (Fig. 4). The patients’ satisfaction with this technique’s outcomes appears to be good, as Group B reported a significantly higher patient satisfaction score, compared to Group A (p < 0.001). Furthermore, no patients in Group B required a secondary touch-up procedure to improve the upper-pole contour, although 15 patients (15.66 %) in Group A underwent liposuction to improve the upper-pole contour; this difference was statistically significant (p = 0.002).

## Conclusion

Based on the vascular anatomy of the pedicled TRAM flap, we were able to selectively salvage zones 2 and 4, which reduced the incidence of fat necrosis to 7.55 % (compared to 20.48 % for the conventional method). In addition, by considering the three-dimensional shape of the natural breast when performing the flap insetting, we achieved superior aesthetic outcomes (a significant increase in the patients’ satisfaction score, from 7.01 ± 0.94 to 8.02 ± 0.82) that did not require secondary touch-up procedures for the upper-pole contour.
